# Disposal rate in different age groups of Karan Fries (Crossbred) males in organized herd

**DOI:** 10.14202/vetworld.2015.192-196

**Published:** 2015-02-19

**Authors:** Achun Panmei, A. K. Gupta, P. R. Shivahre, M. Bhakat, K. Mahesh Singh

**Affiliations:** Dairy Cattle Breeding Division, National Dairy Research Institute, Karnal, India

**Keywords:** culling, disposal, karan fries male, mortality, organized herd

## Abstract

**Aim:**

The present study was carried out to analyze the disposal rate in different age groups of Karan Fries (KF) males in National Dairy Research Institute herd.

**Materials and Methods:**

Records on 1740 KF crossbred bulls born during the period 1997-2012 were collected with an objective to ascertain the effect of genetic and non-genetic (Period of birth and season of birth) factors on the disposal pattern of KF males. The percent of animals disposed from the herd due to mortality and culling was calculated by proportion using descriptive statistics. The data were subjected to Chi-square test to test the difference due to different factors.

**Results:**

Overall disposal rate for the different age groups of 0-1 m, 1-2 m, 2-3 m, 3-6 m, 6-18 m, 18 m-3 year and 3-5 year were calculated as 17.9, 16.3, 14.2, 25.8, 49.0, 37.6 and 51.65%, respectively. In the age groups, 3-6 m, 6-18 m and 3-5 year, effect of periods of birth were found to be statistically significant (p<0.01) for overall disposal rate. Across different seasons of birth, overall disposal rates differed significantly (p<0.01) in different age group except in 3-5 year age group. Differences in overall disposal rate due to genetic group were statistically significant (p<0.01) in 1-2 m, 2-3 m, 3-6 m, 6-18 m, 18-3 year and 3-5 year age groups.

**Conclusion:**

Overview of the results indicated that higher overall disposal rate in age group of 1 month was due to mortality while, in the age groups of >1 month, culling was the primary cause.

## Introduction

Disposal pattern of animal includes culling and mortality together. Culling is the process of removing inferior individuals or breeding animals from the herd based on specific criteria, whereas mortality is the phenomenon, which is occurring in the farm due to various factors. Criteria for disposal of male animals from the herd may be based on population, pedigree performance, growth and reproduction causes (libido, semen quality) depending on the goal of breeding program. To accelerate the pace of genetic progress in a breeding program, knowledge of disposal rate becomes an important tool to improve the breeding stocks through selective breeding based on this information. High rates of disposal due to death and culling result in economic losses to the animal breeders and dairy producers and it may be an indicator of compromised management condition.

Success of livestock production depends on wellbeing of the animals in every stages of life as high disposal rate due to death or culling affects availability of high genetic merit bulls required for test mating which results in low genetic gain, decrease intensity of selection from bull to bull and bull to cow path. Khan *et al*. [1] estimated calf mortality of Punjab Government, farms as 17.98%. Normally mortality rate is higher in younger age group up to 3 months [2]. Males (17.86%) are at higher risk at mortality than female (2.13%) as reported by Banger *et al*. [3], where in contrary, Kinjavdekar *et al*. [4] reported higher female calf mortality than male calf. In India, researchers have reported mortality rate of 7.21-17.12% in cattle under organized and field conditions [5]. Therefore, this study was carried out with an objective to ascertain the effect of genetic and non-genetic factors on the disposal rate of different age groups of Karan Fries (KF) males.

## Materials and Methods

### Ethical approval

There was no sacrifice or animal experimentation involved in the present study therefore approval from Institutional Animal Ethics Committee was not required. However, there was no deviation from rules of ethical treatment to animals during the study.

### Data

Data on 1740 KF (Holstein Friesian × Tharparkar) males, born during the period 1997-2012, were collected from records available in Dairy Cattle Breeding record room and Livestock Research center, National Dairy Research Institute (NDRI), Karnal, Haryana. The place is located at an altitude of 250 m above the mean sea level on 29.430 N latitude and 72.20 E longitudes. A subtropical type of climate prevails in the farm area. Four major seasons are prevalent across the year *viz*. Winter (December to March), summer (April to June), rainy (July to September) and autumn (October and November). The area experiences a maximum and minimum temperature of approximately 46°C and 0°C in summer and winter months respectively. The average annual rainfall are received mostly during the month of July and August (760 mm to 960 mm approximately). Relative humidity ranges from 41% to 91%. To estimate the overall disposal rate, records on KF males were categorized into seven age groups *viz*., 0-1 m, 1-2 m, 2-3 m, 3-6 m, 6-18 m, 18 m-3 year and 3-5 year. To assess the effect of various genetic and non-genetic factors on disposal rate of KF males, the data were classified into periods of birth (P-1, 1997-2000; P-2, 2001-2004; P-3, 2005-2008 and P-4, 2009-2012), seasons of birth (S-1, winter; S-2, summer; S-3, rainy and S-4, autumn) and genetic groups (G-1, F1; G-2, Interbred; and G-3, > ¾ crosses). The percent of animals disposed from the herd due to mortality and culling was calculated by proportion. To study the effect of period of birth, season of birth and genetic groups on disposal rate in different age groups of male, Chi-square value was estimated. Data were analyzed using MS-Excel spread sheets.

## Results and Discussions

The overall disposal rate of different age groups of KF males is presented in Table-1. The percentage of disposal in KF males found in the present study was higher than the finding of Sethi *et al*. [6] in KF males while culling rate was comparable with that reported by Mathew *et al*. [7] in crossbred bulls. Overview of the results indicated that higher overall disposal rate up to 1 month of age was due to mortality, while above 1 months of age group, it was primarily due to culling (surplus, undesirable animals, poor semen quality, etc.). Shivahre *et al*. [8] have reported mortality rate (17.49) in Murrah males of 0-1 m age group, which was higher than our present findings. Overall disposal rate was highest in 3-5 year age group, which was primarily due to culling of surplus breeding bulls as the production target of the bull has achieved. Chauhan [9] conducted a study on KF males at NDRI herd and revealed that important cause of disposal for male calves below 1.5 year of age are mortality and in case of above 1.5 year of age group, culling due to poor growth and poor semen quality were the main reasons. Taraphder *et al*. [10] analyzed disposal pattern in an organized herd of Murrah buffalos and found that total disposal rate was 26.10% with 20.77% culling and 5.33% mortality in adult buffaloes. Bangar *et al*. [3] conducted a study on mortality in cattle covering village areas of Pune division of Maharashtra state and reported that an overall mortality rate in cattle with 16.81%, 1.46% and 0.76% in <1 year, 1-3 year and >3 year age groups respectively, which was lesser than our present findings. Katoch *et al*. [11] reported that half of the mortality in calves occurred in the first 15 days of life. Pradhan and Panda [12] also observed that high mortality in Murrah buffalo calves occurred mainly during 0-3 months of age while Singh *et al*. [13] estimated overall mortality to be more (45.8%) in 0-6 months age group for crossbred calves.

**Table-1:** Disposal rate in different age group of KF males.

Age group	No. available	Culling rate	Mortality rate	Disposed rate
0-1 m	1740	129 (7.4)	183 (10.5)	312 (17.9)
1-2 m	1428	144 (10.1)	88 (6.2)	232 (16.2)
2-3 m	1196	111 (9.8)	59 (4.9)	170 (14.2)
3-6 m	1026	203 (19.7)	62 (6.0)	265 (25.8)
6-18 m	761	277 (36.1)	96 (12.6)	373 (49.0)
18 m -3 year	388	140 (36.1)	6 (1.5)	146 (37.6)
3-5 year	242	117 (48.3)	8 (3.3)	125 (51.7)

Figures in parentheses indicate %, KF=Karan Fries

The effect of genetic groups on disposal of KF males of different age groups is shown in Table-2. In genetic group G1, a few numbers of animals were available in each age group. Disposal in younger males of age group 1-6 m was contributed equally by culling and mortality. On the other hand, culling was the sole cause of disposal in males of age group 6 m-5 year. In genetic groups G2, males in different age groups were disposed due to culling, except in age group of 0-1 m, which was due to mortality. In G3, mortality was the main reasons of disposal up to age group 18 m while culling was the main cause of disposal in older age group males with 6-18 m age group males having the highest disposal rate. The results revealed that males in G2 genetic group had the highest (88.79%) overall disposal rate due to culling while, G2 and G3 had low disposal rate of (2.71%) and (8.50%), respectively. Differences in overall disposal rate due to genetic group were statistically significant (p<0.01) in 1-2 m, 2-3 m, 3-6 m, 6-18 m, 18-3 year and 3-5 year age groups.

**Table-2:** Effect of genetic group on disposal rate of different age group of KF males.

Effects	0-1 m	1-2 m	2-3 m	3-6 m	6-18 m	18 m-3 year	3-5 year	Overall disposed
Genetic group								1623
G1								
No. available	48	45	41	41	37	33	26	
Mortality	3 (6.25)	2 (4.44)	0	2 (4.88)	0	0	0	
Culling	0	2 (4.44)	0	2 (4.88)	4 (10.81)	7 (21.21)	22 (84.62)	
Disposal	3 (6.25)	4 (8.89)	0	4 (9.76)	4 (10.81)	7 (21.21)	22 (84.62)	44 (2.71)
G2								
No. available	1530	1242	1019	859	617	278	158	
Mortality	163 (10.65)	84 (6.76)	51 (5.00)	47 (5.47)	80 (12.97)	5 (1.80)	8 (5.06)	
Culling	125 (8.17)	139 (11.19)	109 (10.70)	195 (22.70)	259 (41.98)	115 (41.37)	61 (38.61)	
Disposal	288 (18.82)	223 (17.95)	160 (15.70)	242 (28.17)	339 (54.94)	120 (43.17)	69 (43.67)	1441 (88.79)
G3								
No. available	162	141	136	126	107	77	58	
Mortality	17 (10.49)	2 (1.42)	8 (5.88)	13 (10.32)	16 (14.95)	1 (1.30)	0	
Culling	4 (2.47)	3 (2.13)	2 (1.47)	6 (4.76)	14 (13.08)	18 (23.38)	34 (58.62)	
Disposal	21 (12.96)	5 (3.55)	10 (7.35)	19 (15.08)	30 (28.04)	19 (24.68)	34 (58.62)	138 (8.50)
*χ*^2^ value for disposal rate	8.68*	24.21**	13.94**	16.24**	49.92**	12.92**	17.17**	

Figures in parentheses indicate %,

* *χ*(p<0.05),

** *χ*(p<0.01), KF=Karan Fries

The effect of period of birth and season of birth on disposal of KF males of different age groups are presented in Table-3. It was observed that for the periods P1 and P2, the maximum disposal rate was found in the age group 3-5 year while in P3, highest disposal rate was found in the age group 6-18 m. However, minimum disposal rate was found in the age group of 1-2 m for P1 (16.90%) and 2-3 m for the period P2 (14.52%) and P3 (14.19%). In P1, P2 and P3 period, culling was the main reason of the disposal for all the age group except in 0-1 m age group of P1, for which mortality was the main cause of disposal. The overall disposal rate in P4 period for the age groups, 18 m-3 year and 3-5 year were not calculated as many of the bulls born during the particular period may not have reached this age yet. Therefore, only P1, P2 and P3 periods were considered for the age group 18 m-3 year and 3-5 year. Overall, better performance in the last two periods P3 (25.42%) and P4 (19.25%) may be due to better feeding and management practices in that period. Nor *et al*. [14] reported that the average culling rate for slaughter/death was 25.4% in Dutch dairy herds over the years from 2007 to 2010. Chaudhary *et al*. [15] conducted a study on morbidity and mortality rates of bovine in Himachal Pradesh for a period of 1 year (January 2011 to December 2011) and reported that overall mortality rate in bovine was 9.14% with calves showing highest mortality of 21.53% followed by young stocks 9.35% and adults 4.73%. In the age groups 3-6 m, 6-18 m and 3-5 year, effect of periods of birth were found to be statistically significant (p<0.01) for overall disposal rate which may be due to different manage mental practices over the years. Across the seasons of birth, disposal rate was highest in the age group 3-5 year mainly due to culling except in S1 where 6-18 m age group revealed the maximum disposal rate. Minimum disposal rate in S1 (16.00%) and S3 (5.52%) were observed in 3-6 m age group, whereas in S2 (7.65%) and S4 (4.78%) it was found in age group of 2-3 m. It was also observed that males of 0-1 m (11.09%) and 3-6 m (3.23%) age group born in winter and rainy seasons were disposed mainly due to mortally, whereas culling was the main reasons of disposal in the remaining age groups. Summer and Autumn born males of 0-1 m, 1-2 m and 2-3 m age group were disposed mainly due to mortality while culling was the cause of disposal above 3 m age group. Babcock *et al*. [16] have reported that there was a seasonal pattern in culling and mortality risk in summer (March-September) than in winter (November-February). Across different seasons of birth, overall disposal rates differed significantly (p<0.01) in different age group except in 3-5 year age group. Cheryl *et al*. [17] reported that the incidence of culling was greatest in fall, followed by winter, then spring, and summer whereas mortality was higher in spring than in summer, fall, and winter. Prasad *et al*. (2004) reported average mortality rate, in organized herd as 4.53%, 4.81% and 4.84% in hot dry (March-June), hot-humid (July-October) and cold (November-February) seasons, respectively.

**Table-3:** Effect of period of birth and season of birth on disposal rate of different age group of KF males.

Effects	0-1 m	1-2 m	2-3 m	3-6 m	6-18 m	18 m-3 year	3-5 year	Overall disposed
Period of birth								1605
P1								
No. available	447	355	295	241	176	89	60	
Mortality	57 (12.75)	30 (8.45)	14 (4.75)	9 (3.73)	15 (8.52)	2 (2.25)	7 (16.67)	
Culling	35 (7.83)	30 (8.45)	40 (13.56)	56 (23.24)	72 (40.91)	27 (30.34)	35 (83.33)	
Disposal	92 (20.58)	60 (16.90)	54 (18.31)	65 (26.97)	87 (49.43)	29 (32.58)	42 (70.00)	429 (26.73)
P2								
No. available	470	375	310	265	223	151	76	
Mortality	44 (9.36)	13 (3.47)	11 (3.55)	9 (3.40)	13 (5.83)	1 (0.66)	0.00	
Culling	51 (10.85)	52 (13.87)	34 (10.97)	33 (12.45)	59 (26.46)	74 (49.01)	65 (100)	
Disposal	95 (20.21)	65 (17.33)	45 (14.52)	42 (15.85)	72 (32.29)	75 (49.67)	65 (85.53)	459 (28.60)
P3								
No. available	432	370	310	266	175	66	42	
Mortality	26 (6.02)	13 (3.51)	12 (3.87)	16 (6.02)	32 (18.29)	2 (3.03)	1 (5.56)	
Culling	36 (8.33)	47 (12.70)	32 (10.32)	75 (28.20)	77 (44.00)	22 (33.33)	17 (94.44)	
Disposal	62 (14.35)	60 (16.22)	44 (14.19)	91 (34.21)	109 (62.29)	24 (36.36)	18 (42.86)	408 (25.42)
P4								
No. available	391	328	281	254	187	82	64	
Mortality	56 (14.32)	32 (9.76)	22 (7.830	28 (11.02)	36 (19.25)	-	-	
Culling	7 (1.79)	15 (4.57)	5 (1.78)	39 (15.35)	69 (36.90)	-	-	
Disposal	63 (16.11)	47 (14.33)	27 (9.61)	67 (26.38)	105 (56.15)	-	-	309 (19.25)
*χ*^2^ value for disposal rate	8.44*	2.05	8.96*	23.74**	41.12**	7.75*	23.56**	
Season of birth								1623
S1								
No. available	667	524	430	350	294	130	77	
Mortality	74 (11.09)	28 (5.34)	25 (5.81)	22 (6.29)	50 (17.01)	1 (0.77)	2 (5.26)	
Culling	69 (10.34)	66. (12.60)	55 (12.79)	34 (9.71)	114 (38.78	52 (40.00)	36 (94.74)	
Disposal	143 (21.44)	94 (17.94)	80 (18.60)	56 (16.00)	164 (55.78)	53 (40.77)	38 (49.35)	628 (38.39)
S2								
No. available	423	369	340	314	164	96	62	
Mortality	53 (12.53)	26 (7.05)	17 (5.00)	25 (7.96)	14 (8.54)	2 (2.08)	3 (7.50)	
Culling	1 (0.24)	3 (0.81)	9 (2.65)	125 (39.81)	54 (32.93)	32 (33.33)	37 (92.50)	
Disposal	54 (12.77)	29 (7.86)	26 (7.65)	150 (47.77)	68 (41.46)	34 (35.42)	40 (64.52)	401 (24.71)
S3								
No. available	405	307	217	163	154	91	67	
Mortality	39 (9.63)	15 (4.89)	7 (3.23)	5 (3.07)	10 (6.49)	2 (2.20)	1 (3.57)	
Culling	59 (14.57)	75 (24.43)	47 (21.66)	4 (2.45)	53 (34.42)	22 (24.18)	27 (96.43)	
Disposal	98 (24.20)	90 (29.32)	54 (24.88)	9 (5.52)	63 (40.91)	24 (26.37)	28 (41.79)	366 (22.55)
S4								
No. available	245	228	209	199	149	71	36	
Mortality	17 (6.94)	19 (8.33)	10 (4.78	10 (5.03)	22 (14.77)	1 (1.41)	2 (10.53)	
Culling	0 (0.00)	0 (0.00)	0 (0.00)	40 (20.10)	56 (37.58)	34 (47.89)	17 (89.47)	
Disposal	17 (6.94)	19 (8.33)	10 (4.78)	50 (25.13)	78 (52.35)	35 (49.30)	19 (52.78)	228 (14.05)
*χ*^2^ value for disposal rate	44.17**	69.21**	54.33**	131.70**	13.84**	9.78*	6.90	

Figures in parentheses indicate %, KF=Karan Fries,

* *χ*(p<0.05),

** *χ*(p<0.01)

## Conclusion

Mortality is highly relevant for the definition of disposal of calves, which are in the age group of 0-1 m but with advancing age, culling was the main cause of disposal for the young or adult KF males. The disposal of KF males depends on various factors that involve the simultaneous action of genetic and non-genetic effects. Considering the higher risk of death among male calves, strategies for environmental improvement, health care, and genetic selection should be developed to avoid or to reduce the risk of mortality. It can be concluded that disposal rate at the farm needs to be minimized by monitoring various genetic and manage mental factors.

## Authors’ Contribution

AKG designed the work. AP conducted study and analyzed the data. MB helped in the compilation of data. PRS and KMS helped in writing and revision of the manuscript. All authors read and approved the final manuscript.
